# A Pilot Study of PSMA PET/CT and MRI Fusion for Prostate Cancer: Software to Replace PET/MRI Hardware

**DOI:** 10.3390/jcm13237384

**Published:** 2024-12-04

**Authors:** Georges Mehawed, Matthew J. Roberts, Jessica Bugeja, Jason Dowling, Kate Stewart, Rivindi Gunasena, Frances Malczewski, Nicholas J. Rukin, Rebecca Murray

**Affiliations:** 1 Herston Biofabrication Institute, Metro North Health, Herston, QLD 4029, Australia; nickrukin@hotmail.com (N.J.R.); becca.murray@health.qld.gov.au (R.M.); 2 Urology Department, Redcliffe Hospital, Metro North Health, Redcliffe, QLD 4020, Australia; 3 School of Medicine, University of Queensland, Herston, QLD 4029, Australia; m.roberts2@uq.edu.au; 4 Australian Institute of Bioengineering and Nanotechnology, University of Queensland, St. Lucia, QLD 4067, Australia; 5 Urology Department, Royal Brisbane and Women’s Hospital, Metro North Health, Herston, QLD 4029, Australia; 6 University of Queensland Centre for Clinical Research, University of Queensland, Herston, QLD 4029, Australia; 7 Commonwealth Scientific and Industrial Research Organisation, Australian E-Health Research Centre, Herston, QLD 4029, Australia; jess.bugeja@csiro.au (J.B.); jason.dowling@csiro.au (J.D.); 8 Information Technology and Electrical Engineering, University of Queensland, St. Lucia, QLD 4067, Australia; 9 Department of Radiation Oncology, Royal Brisbane and Women’s Hospital, Metro North Health, Herston, QLD 4029, Australia; kate.stewart@health.qld.gov.au; 10 Department of Radiology, Royal Brisbane and Women’s Hospital, Metro North Health, Herston, QLD 4029, Australia; rivindi.gunasena@health.qld.gov.au; 11 Department of Pathology, Royal Brisbane and Women’s Hospital, Metro North Health, Herston, QLD 4029, Australia; frances.malczewski@health.qld.gov.au

**Keywords:** image fusion, image registration, PSMA PET, MRI, prostate cancer

## Abstract

**Introduction**: Prostate-specific membrane antigen positron emission tomography/computed tomography (PSMA PET/CT), in combination with magnetic resonance imaging (MRI), may enhance the diagnosis and staging of prostate cancer. Image fusion of separately acquired PET/CT and MRI images serve to facilitate clinical integration and treatment planning. This study aimed to investigate different PSMA PET/CT and MRI image fusion workflows for prostate cancer visualisation. **Methods**: Eighteen patients with prostate cancer who underwent PSMA PET/CT and MRI prior to radical prostatectomy were retrospectively selected. Alignment of the prostate was performed between PET/CT and MRI via three techniques: semi-automatic rigid, automatic rigid, and automatic non-rigid. Image fusion accuracy was evaluated through boundary and volume agreement, quantified by the Dice Similarity Coefficient (DSC), 95% Hausdorff Distance (HD), and Mean Surface Distance (MSD), with comparison against reconstructed histopathology slices. **Results**: Image fusion using all techniques resulted in clear lesion visualisation from PSMA PET/CT overlay and anatomical detail afforded by the MRI base and was consistent with histopathology tumour location. Image fusion accuracy was within the recommended range based on a DSC of 0.8–0.9. The automatic non-rigid registration method had the highest volume agreement (DSC: 0.96 ± <0.01) and boundary agreement (HD: 1.17 ± 0.35 mm) when compared to automatic rigid (DSC 0.88 ± 0.02, HD 3.18 ± 0.29 mm) and semi-automatic rigid (DSC 0.80 ± 0.06, HD 5.25 ± 1.68 mm). **Conclusions**: Image fusion of clinically obtained PET/CT and MRI is feasible and clinically acceptable for use in prostate cancer diagnosis and surgical management. While the best accuracy was observed with the automatic non-rigid technique, which requires further validation, image fusion with clinically accessible methods (semi-automatic rigid) may currently aid patient education, pre-operative planning, and intra-operative guidance.

## 1. Background

Prostate cancer is the second most commonly diagnosed cancer in men worldwide with multiparametric magnetic resonance imaging (mpMRI) being an established diagnostic tool, especially to guide biopsy targeting [1,2]. Prostate-specific membrane antigen positron emission tomography computed tomography (PSMA PET/CT) is being increasingly utilised in primary staging, while the additional benefit of improved sensitivity in primary tumour detection is of interest [3,4,5]. When combined with MRI, PSMA PET has improved negative predictive value for diagnosing clinically significant prostate cancer [6]. Despite these clinical advantages, both imaging modalities are usually still reviewed separately in clinical practice.

Hybrid PET/MRI can provide both diagnosis and staging in one instance; however, due to a worldwide paucity of PET/MRI scanners and their technical limitations, most clinicians and patients do not have access to hybrid PSMA PET/MRI imaging [3,7,8]. Image fusion is a technique that involves combining the critical information from the two sets of imaging modalities. An example of fusion in urology is ultrasound and MRI for prostate biopsy, which is increasingly utilised, as it showed improved visualisation of lesions identified on MRI [9]. Fused PSMA PET/CT and MRI images are commonly used by radiation oncologists for the radiation planning of prostate cancer treatment [10,11].

Data supporting the image fusion of PSMA PET and MRI are lacking and so it is not routine in urological practice; instead, a separate inspection is performed at the expense of confidence in lesion detection, especially for discordant findings and multifocal disease. Fused PET and MRI may then have a future role in prostate biopsy guidance, as well as pre-operative planning and intra-operative guidance for radical prostatectomy [12]. Although improving the sensitivity of prostate target biopsies [13] and reducing the positive surgical margin in radical prostatectomy [14,15] are areas of interest, there are no studies to our knowledge that have explored the role of the image fusion of PSMA PET/CT and MRI in urological surgery. The combination of PSMA PET/CT and MRI better correlates with histopathology, improves the detection of multifocal disease, and thus may enhance visualisation, which in turn may lead to better diagnosis and treatment outcomes [3,7,11].

The aim of this pilot study was to investigate combining PSMA PET/CT and MRI through image fusion by (1) demonstrating clinical and non-clinical image fusion workflows and (2) exploring whether image fusion of PSMA PET/CT and MRI could enhance visualisation through improved prostate tumour *detection* and *localisation*. Tumour visualisation was compared in terms of the accuracy of *detection* (tumour present or absent) and *localisation* (fusion/registration accuracy and comparison of fused images to histopathology).

## 2. Methods

Institutional ethics approval was obtained to investigate workflows and accuracy measures for PSMA PET/CT and MRI image fusion. Eighteen patients who underwent mpMRI followed by transperineal prostate biopsy and PSMA PET/CT prior to radical prostatectomy were retrospectively selected, from an already existing local database containing MRI and PSMA PET information for prostate cancer detection, to represent a mixture of PET and MRI findings as outlined in three scenarios:

1. MRI positive (present) and PSMA PET/CT positive (present);
2. MRI positive (present) and PSMA PET/CT negative (absent);
3. MRI negative (absent) and PSMA PET/CT positive (present).

*Detection* of prostate tumours was defined as either absent or present on the imaging modalities based on the imaging report.

Clinical details including age, prostate-specific antigen (PSA) levels, imaging findings, radical prostatectomy histology report, and whole-mount specimen photos were recorded.

### 2.1. Imaging Acquisition

Separate mpMRI and PSMA PET/CT scans, acquired as part of routine clinical care, were reviewed. PET/MRI was not performed in this cohort. mpMRI scans were obtained with 3 Tesla Skyra (Siemens, Germany, 0.5 mm × 0.5 mm × 3 mm slice resolution) without an endorectal coil according to well-established mpMRI detection protocols [16]. Images included T2 weighted imaging (T2WI), axial apparent diffusion coefficient (ADC) values, diffusion weighted imaging (DWI), T1 weighted imaging (T1WI), and T1WI post-contrast imaging. DWI was obtained by using b-value of 2000 s/mm^2^. Dynamic acquisition was performed with 8 mL of Gadovist (gadolinium-based contrast agent) at a rate of 3 mL/s with a temporal resolution of 2 min and 21 s. Particular interest was given to T2WI, ADC, and DWI sequences in the axial plane. mpMRI images were interpreted and reported by experienced uro-radiologists using the Prostate Imaging Reporting and Data System (PI-RADS) transitioning from version 1 to version 2 in 2016 [17]. In this study, lesions/areas reported as PIRADS 1–3 were defined as absent and PIRADS 4–5 were defined as present.

Ga-68 PSMA PET/CT scans were obtained using a Siemens Biograph mCT (Siemens, Munich, Germany, 0.5 mm × 0.5 mm × 1 mm slice resolution) and images were obtained from the skull vertex to the thighs following the intravenous injection of 150 MBq +/− 5% of Ga-68 ligand (HBED-CC urea-based small-molecular inhibitor of PSMA, “Ga-68 PSMA”) with an uptake time of 45 min, manufactured at the Specialised PET Services Queensland Radiopharmaceutical Laboratory [18]. A low-dose CT scan was performed during tidal respiration for attenuation correction and lesion *localisation*. Images were interpreted and reported by experienced nuclear medicine specialists, and lesions were defined as present if PSMA uptake (measured by maximum standardised uptake value—SUVmax) was significantly higher than surrounding prostatic tissue or absent/equivocal if PSMA uptake was marginally higher.

### 2.2. Histopathology

Radical prostatectomy specimens were evaluated and reported by uro-pathologists using the International Society of Urological Pathology (ISUP) protocols for Gleason grading of prostatic carcinoma [19]. Index tumours were defined as the lesion with the highest Gleason score [20].

Histology slides were acquired and combined to digitally construct axial slices of the prostate (microscope: Axio Observer, ZEISS, Jena, Germany; software: ZEN 2.3 (blue edition), ZEISS, Jena, Germany).

### 2.3. Image Fusion Techniques

Prior to image fusion, manual segmentation of whole prostate volume (PV) on the CT component of the PET/CT and the T2WI of the MRI was performed on MIM Maestro (Version 7.6.1, MIM Software, Beachwood, OH, USA) and verified by a radiologist. These manual segmentations were required for performing automatic registration methods, calculating accuracy measures for all registration methods, and enabling further comparisons through 3D volume and model construction.

**Figure A1:**
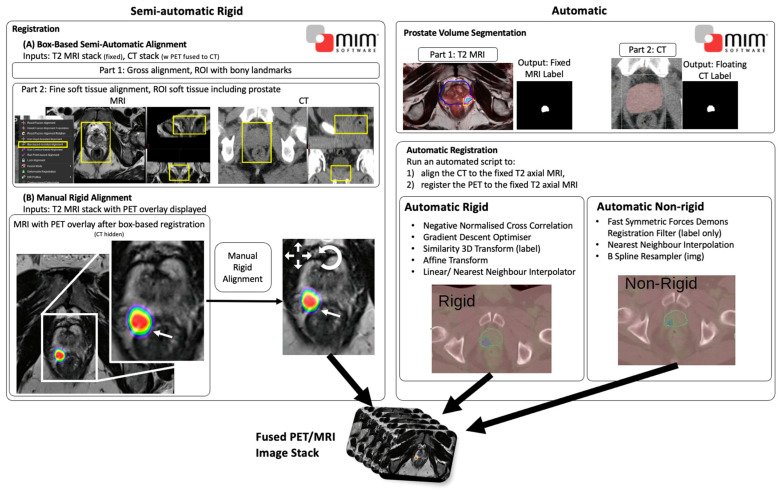
Semi-automatic rigid and the two automatic registration workflows used to fuse the PET image data onto the T2 axial MRI data set.

Image fusion (Figure A1) was performed using three methods: (1) *semi-automatic rigid* (only image rotation and translation allowed), (2) *automatic rigid*, and (3) *automatic non-rigid* (rotation, translation, and localised stretching of images allowed). For all three registration techniques, the axial T2WI MRI sequence was fixed and the overlaid CT image floated to position corresponding to MRI landmark. The windowing for SUVmax range was set from 4 to 12 since an SUVmax of 4 has high sensitivity (92%) in detecting clinically significant prostate cancer, an SUVmax < 4 was considered negative for prostate cancer, and an SUVmax of 12 had a 100% specificity and positive predictive value [6]. Bugeja et al. describe all three registration techniques [21].

### 2.4. Accuracy and Statistical Measures

Image registration accuracy measures were calculated to provide quantitative measures to compare the *localisation* accuracy of the image fusion methods. Contour, volume, and boundary agreement testing were performed and reported using the typical measures of 95% Hausdorff Distance (HD), Mean Surface Distance (MSD), and Dice Similarity Coefficient (DSC). Paired *t*-tests were used to compare the volume and boundary overlap between the semi-automatic rigid, automatic rigid, and automatic non-rigid registration techniques. Statistical significance was set at *p* < 0.05.

*Localisation* of the image fusion methods was also visually compared against the “ground truth” histopathology, which included the report, a comparable axial pathology specimen image, and digitally constructed axial histology slices. Location and volume of tumours were compared between the fused images and the histopathology report, pathology surgical specimen slices, and multi-slide digitally reconstructed histology slices.

## 3. Results

### 3.1. Lesion Detection

All selected patients underwent robotic-assisted laparoscopic prostatectomy (RALP) for histologically confirmed prostate cancer (Table 1). A consistent diagnostic pathway was used, including MRI followed by prostate biopsy, then staging with PSMA PET/CT prior to RALP. The mean time between MRI and PSMA PET/CT was 3 months and 1.6 months between PSMA PET/CT and RALP.

All patients had a varying index tumour *detection*. Imaging findings were reported as either present or absent for the lesion of interest in each patient. Included patients had a variety of concordant (n = 9) and discordant (n = 9) disease (Table 1). Although the index tumour was not detected by MRI in MRI−/PET+ patients, all index tumours were detected in the fused PSMA PET/CT and MRI images in all three image fusion methods.

### 3.2. Lesion Localisation

**Figure 1:**
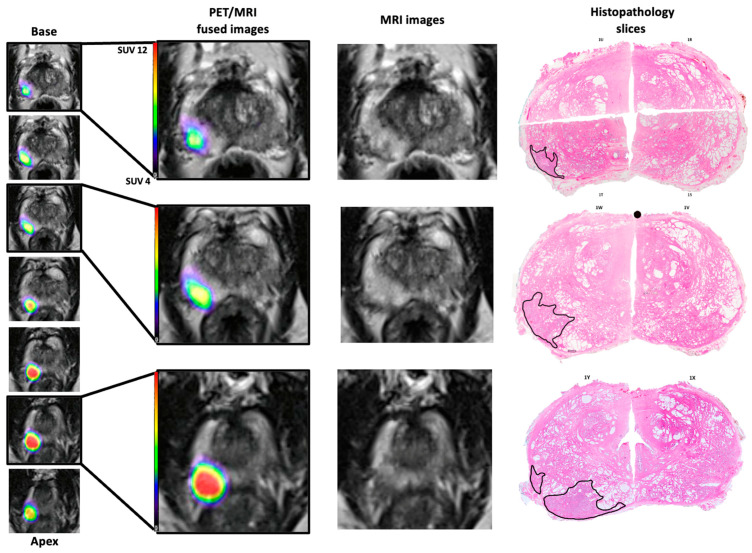
PET/MRI fused images qualitatively compared to T2WI axial slices of MRI and digitally recreated histopathology slices for a patient from the MRI−/PET+ group. Note the similar localisation of the tumour: right posterior from base to apex. The histology slices are digitally recreated from multiple histology slides and the tumour outlines (black) performed by an experienced uro-pathologist. Note the spacing between the fused images is 3 mm whereas the histopathology slides are approximately 10 mm.

When comparing the histopathology results to the fused images, the tumour location appeared consistent between the uro-pathologist report, the constructed axial histology slices, and the fused images (Figure 1). In one patient from the MRI−/PET+ group, with unfavourable intermediate-risk prostate cancer, the index lesion was concordantly located in the right posterior peripheral zone extending from base to apex, which is clearly seen on the constructed histology slices and fused PET/MRI images (Figure 1). Similarly, in the MRI+/PET+ group, lesions were accurately located by fused, MRI, PET, and histopathology.

### 3.3. Workflow Accuracy Measures

**Figure 2:**
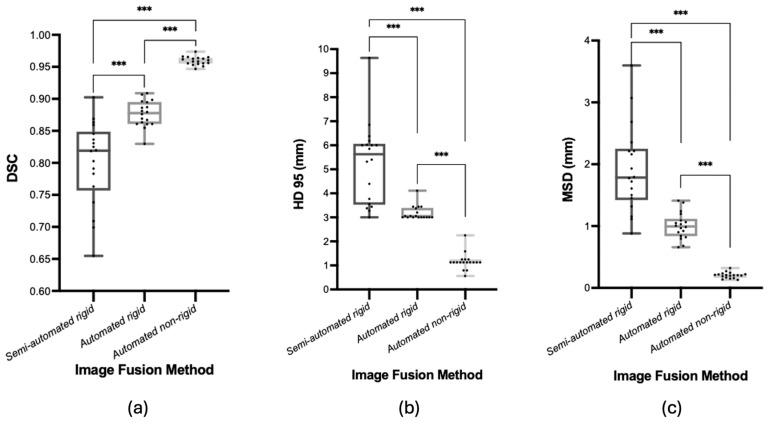
Boxplots of boundary and volume agreement for the image fusion methods used between PSMA PET/CT and MRI—(**a**) Dice Similarity Coefficient, (**b**) 95% Hausdorff Distance, (**c**) Mean Surface Distance. Lines across boxplots represent the median value. *** represents *p*-value of <0.001.

Although the automatic methods were quicker, all methods completed image fusion in around 5 min or less. The accuracy of image fusion was found to be best for all measures using the *automatic non-rigid* registration, followed by *automatic rigid,* then *semi-automatic* (Figure 2). Volume agreement calculated by DSC was highest at 0.96 ± <0.01 for *automatic non-rigid* compared to 0.88 ± 0.02 for *automatic rigid* and 0.80 ± 0.06 for *semi-automatic rigid* registrations (*p* < 0.001). Boundary agreement was also highest for *automatic non-rigid* (MSD: 0.20 ± 0.05 mm) compared to *automatic rigid* (MSD: 1.00 ± 0.21 mm) and *semi-automatic rigid* (MSD: 1.92 ± 0.70 mm) (*p* < 0.001). The 95% Hausdorff Distance represents the distance of the worst alignment, and the *automatic non-rigid* method (1.17 ± 0.35 mm) had the smallest discrepancy compared to *automatic rigid* (3.18 ± 0.29 mm) and *semi-automatic rigid* (5.25 ± 1.68 mm) (*p* < 0.001).

## 4. Discussion

Image fusion of PSMA PET/CT and MRI serves to improve the visualisation of intraprostatic tumours for diagnostic and treatment purposes. However, to be a viable clinical tool for clinicians, it must be clinically available, not overly time-consuming, and provide enhanced utility. This study compared three varying methods of image fusion for prostate cancer and found that while the *automatic non-rigid* technique was the most accurate, the *semi-automatic rigid* workflow was accessible and provided enhanced visualisation compared to a single image modality through improved *detection* and acceptable *localisation*.

The addition of PSMA PET/CT to MRI has growing evidence in enhancing diagnosis and primary staging, where the combination of both PSMA PET and MRI leads to improved lesion detection when compared to MRI alone but also improved prediction of lymph node involvement when compared to PSMA PET/CT alone [5,6,22,23]. PSMA PET/CT and MRI image fusion was achieved by overlaying the PSMA PET/CT axial series on top of the axial T2WI MRI base image. The result was clearly visible tracer avidity of prostate tumours (from the PSMA PET) combined with the high anatomical detail of the prostate, fascial planes, neurovascular bundle, and surrounding structures (seminal vesicles, bladder, and rectum), which were maintained with the MRI base image (Figure A1). The combined anatomic detail and lesion avidity improved both *detection* and *localisation*, and could be viewed within one image stack, which was concordant with the ground truth (histopathology). Image fusion of PSMA PET/CT and MRI has increased sensitivity of index lesion identification (*detection*) and *localisation*, comparable with hybrid PET/MRI [7,24]. Image fusion is a viable alternative to hybrid PET/MRI given that PSMA PET/CT is more accessible and funded federally in Australia [25], and hybrid PET/MRI scanners are more expensive and scarce [8].

Thus, it is believed that fused images could achieve the benefits of hybrid PET/MRI including superior diagnostics (such as adding PSMA PET to diagnostic paradigms in patients with negative MRI but high degree of clinical suspicion for prostate cancer leading to improved detection) [6,26] and impact on altering management (such as prostatectomy approach and planning including nerve sparing and prediction of positive surgical margins relative to adjacent or at-risk structures) [3,22]. For example, biopsy guidance through the cognitive fusion of fused images may be able to mimic the performance of hybrid PSMA PET/MRI in its high accuracy of detecting clinically significant prostate cancer [27].

**Figure 3:**
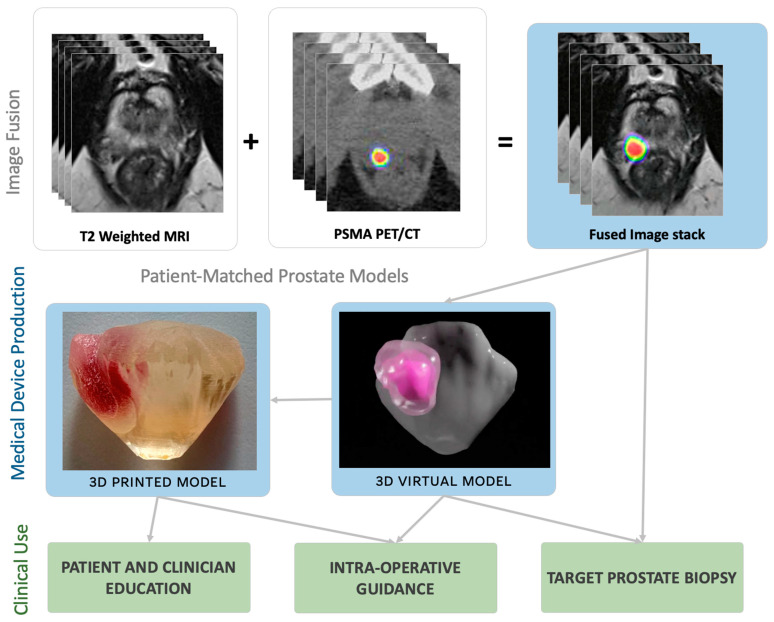
Clinical utility pathways for applying the fused PET/MRI image fusion in urological practice.

Given the known benefits of hybrid PET/MRI, the demonstrated enhanced visualisation through the fused images, and the clinical integration and accessibility of the fused images (seen in radiation oncology), we believe fused images can have their application extended to provide further utility in prostate cancer diagnosis and management via 3D models. Three-dimensional virtual and printed models can be created based on the fused image stack of PSMA PET/CT and MRI. These 3D models may be used for improved patient education and experience [28], and intra-operative guidance in spatially improved cognitive fusion prostate biopsy and robotic-assisted radical prostatectomy (Figure 3). Intra-operative guidance with 3D models, based on MRI alone, in radical prostatectomy has shown a reduction in positive surgical margins [14,15]. Given that multifocal disease has a higher risk of positive surgical margins and that fused PET and MRI can increase the *detection* of bilateral and multifocal disease [7,29], 3D models based on the fused stack may enhance intra-operative guidance in view of reducing the risk of a positive surgical margin.

Three image fusion workflows were displayed in this study. The *semi-automatic rigid* image fusion process on MIM Maestro is currently a clinically available pathway used by radiation oncologists. It offers a built-in semi-automatic alignment but may require manual rigid alignment adjustments often due to variable natural bladder/rectal filling, different imaging protocols including bladder filling and endorectal coils, and soft tissue deformation of the prostate. This method is appealing for its clinical availability since it is already in use by radiation oncology for radiation planning, dose painting, and delivery of focal radiation due to the improved biological characterisation through the combination of PSMA PET and MRI [10]. When compared to *automatic* registrations, *semi-automatic rigid* registration requires less than 5 min to perform through an experienced health professional but does not require the often-time-consuming PV segmentation prior to image fusion. Since it can be performed on software already available in hospital systems, the main cost is time including image transfer from different systems and the health professional performing the fusion, which may be substantial in a stretched healthcare system.

*Semi-automatic* registration was overall less accurate than the *automatic* methods with a DSC of 0.80 compared to DSC values of 0.88 and 0.96, where it was the only method requiring manual alignment to improve registration qualitatively. Given that PV registration in this study was between MRI and the CT component of PET/CT, the *semi-automatic* accuracy was similar to the clinically reported ranges of DSC for PV registration between a different modality fusion (MRI and CT) (0.72–0.79 [30,31]) and the same modality fusion (MRI or CT) (~0.8–0.9 [32]). PV is shown to vary between MRI and CT, where CT volumes can be 1.4 times larger than MRI volumes [33]. *Rigid* registration does not perform any localised image deformation; thus, lower DSC values are expected for fusion between the different modalities. Given the current clinical accessibility of the semi-automatic technique and the demonstrated registration accuracy (DSC 0.8), we believe the resulting fused images could immediately be clinically trialled to test for possible clinical benefits.

The future lies in the automation of the workflow for a reduction in time and error, but the automatic methods described here would only be clinically useful if automatic PV segmentation were feasible, and such techniques are emerging [34,35,36]. *Automatic*, *rigid,* and *non-rigid* registration methods are promising as they demonstrate high accuracy quantitively in both volume and boundary agreement, especially the *non-rigid* method (Figure 2). These automatic methods are based on algorithm development and thus are quick once PV segmentations are loaded onto the software. However, they are not quite ready for clinical use as they require more rigorous verification and validation of the clinically applied algorithm and accelerated segmentation methods since manual contouring of PV on MRI and CT is time-consuming and thus limits the technique’s current clinical feasibility. Validating these algorithms would require a larger study with quantitative and qualitative comparisons to hybrid PET/MRI images (gold standard). There are promising examples of automatic PV segmentation methods, which appear acceptable for clinical use but are not yet applied in clinical practice [34,35,36,37]. Regulatory approval is required prior to integrating automatic segmentation methods, and manual review and adjustment remain critical to address cases where automation might underperform due to patient variability or image quality challenges. The advent of *semi-/automatic* PV segmentation that feeds into automatic registration tools could provide an accurate, fast, and clinically relevant fused image stack.

This pilot study is an initial workflow development and assessment study and as such has limitations including its retrospective monocentric design and small sample size. The imaging scans were performed on different days, which may affect prostate and tumour size as well as bladder and rectal filling [38]; however, this reflects normal practice in prostate cancer care. Given that the patients already underwent RALP, image fusion cannot be compared between different PET tracers and their parameters (type of scanner and time from injection to scan), and hybrid PET/MRI scans or ex vivo MRI scans for the co-registration of the pathology specimen to the fused images. The SUV range is not well documented in the literature in the setting of the management of prostate cancer, and a windowing range of 4–12 was used based on a different cohort with the aim of maximising the diagnosis and *detection* of prostate cancer [6]. To justify license and time costs, the use of image fusion in the urological management of prostate cancer can be validated through a larger prospective study prior to being widely adopted.

## 5. Conclusions

In conclusion, image fusion of PSMA PET/CT and MRI using clinical software is currently accessible, accurate (within clinically acceptable range), and can aid the visualisation of prostate cancer for use in diagnosis and management. Current integration of image fusion into clinical practice is possible; however, future research should evaluate the clinical benefits of automatic image fusion integration, potentially through prospective assessment for prostate cancer management from patients’ and urologists’ perspectives.

## Figures and Tables

**Table 1:** Clinical summary for the three patient groups with prostate cancer including imaging and histopathology findings.

	Feature	MRI+/PET+	MRI+/PET−	MRI−/PET+
*Characteristics*	*Number of patients* *Age (mean ± SD) in years*	9 * 64.1 ± 6.2	2 57 ± 9.9	7 63.3 ± 6.6
	*PSA (mean ± SD) in ng/mL*	13 ± 17.5	2.8 ± 2.7	8.9 ± 3.3
	*MRI (PI-RADS score)*	4 (*n* = 2) 5 (*n* = 7)	4 (*n* = 2)	1 (*n* = 1) 2 (*n* = 5) 3 (*n* = 1) #
	*PSMA PET/CT (lesion absent or present)*	Present	Absent	Present
*Surgical pathology*	*ISUP score*	2 (*n* = 1) 3 (*n* = 4) 5 (*n* = 3)	2 (*n* = 2)	2 (*n* = 2) 3 (*n* = 3) 4 (*n* = 1) 5 (*n* = 1)
	*Pathological stage*	pT2 (*n* = 2) pT3a (*n* = 4) pT3b (*n* = 3)	pT2 (*n* = 2)	pT2 (*n* = 3) pT3a (*n* = 3) pT3b (*n* = 1)
	*Positive margins, n(%)*	5 (55%)	1 (50%)	2 (28%)

*Abbreviations: PSA—prostate-specific antigen, MRI—magnetic resonance imaging, PSMA PET/CT—prostate-specific membrane antigen positron emission tomography/computed tomography, PI-RADS—Prostate Imaging Reporting and Data System, ISUP—International Society of Urological Pathology.* * One patient had a PSA of 59 (outlier) and was placed on neoadjuvant androgen deprivation therapy, which led to no Gleason grades/ISUP score assigned for histopathology due to treatment effect. # PI-RADS 3 due to diffuse low signal changes within the prostate most likely due to prostatitis.

## Data Availability

Pooled aggregate data can be accessed on request by corresponding author.
